# Preliminary Functional Analysis of the Gut Microbiome in Colic Horses

**DOI:** 10.3390/ani14223222

**Published:** 2024-11-10

**Authors:** Pamela Thomson, Daniel Garrido, Rodrigo Santibáñez, Felipe Lara

**Affiliations:** 1 Laboratorio de Microbiología Clínica y Microbioma, Escuela de Medicina Veterinaria, Facultad de Ciencias de la Vida, Universidad Andrés Bello, Santiago 7550196, Chile; 2 Departamento de Ingeniería Química y Bioprocesos, Facultad de Ingeniería, Pontificia Universidad Católica de Chile, Santiago 8331150, Chile; dgarridoc@uc.cl (D.G.); rsantibanez.uchile@gmail.com (R.S.); 3 Unidad de Medicina y Cirugía Equina, Hospital Clínico Veterinario, Escuela de Medicina Veterinaria, Facultad de Ciencias de la Vida, Universidad Andrés Bello, Santiago 7550196, Chile; felipe.lara@unab.cl

**Keywords:** gut microbiome, equine, colic

## Abstract

The gut microbiome plays a critical role in maintaining horse health, influencing digestion, immunity, and overall well-being. However, in certain conditions like colic, there is evidence of significant alterations in the microbial community. To analyze the composition of the fecal microbiome and the enriched predicted metabolic functions of horses with colic compared to a control group, 14 horses with colic and 14 control horses were recruited. From a stool sample, DNA extraction was carried out for subsequent 16S rRNA metagenomic analysis. The composition of the microbiome was analyzed from the sequences of each sample using the QIIME version 1.8.0 and DADA2 version 1.22 programs. PICRUSt2 was used to predict metabolic functions. Statistical analyses were performed with the Mann–Whitney U test from the Python scipy v1 package. The gut microbiomes of both groups were dominated by *Firmicuteota*, *Bacteroidota*, and *Pseudomonadota* phyla. Colic in horses was associated with reduced diversity, reduced abundance of *Fibrobacter*, and an increase in *Streptococcus*. The abundance of *Firmicuteota* was negatively correlated with *Pseudomonadota* and *Actinobacteriota.* The equine colic microbiome was predicted to be enriched in aerobic respiration pathways and fatty acid and amino acid degradation. These observations indicate discrete but important differences in the gut microbiome of colic horses.

## Simple Summary

Colic in horses is a common condition that can affect different organs of the abdominal cavity. In recent years, attempts have been made to associate this pathology with changes that occur in the intestinal microbiome. Through a case–control study, we analyzed the intestinal microbiome of a group of healthy horses and another with colic by massive sequencing of 16S rRNA to observe the differences in the bacterial composition and functionality of these groups. The intestinal microbiomes of both groups are dominated by the phyla *Firmicuteota*, *Bacteroidota*, and *Pseudomonadota*. The abundance of *Firmicuteota* was negatively correlated with *Pseudomonadota* and *Actinobacteriota* in horses with colic. The microbiome of equine colic was predicted to be enriched with aerobic respiration pathways and fatty acid and amino acid degradation, observations that indicate discrete but important differences in the intestinal microbiome of horses with colic, which correlate with a more pro-inflammatory microbial community.

## 1. Introduction

The intestinal microbiome of animals is a diverse ecosystem composed mainly of bacteria that co-inhabit with viruses, archaea, and fungi in a symbiotic environment [1]. In horses and other non-ruminant herbivores, fibrinolytic bacteria are largely responsible for fiber fermentation [2,3], resulting in the release of short-chain fatty acids (SCFAs) such as butyrate [4,5]. Beyond fiber digestion, the microbiome contributes to a protection barrier against pathogens and is integral to the development and maintenance of the immune system [6], which has been evidenced through the production of secretory IgA by the host and the composition and production of SCFAs in the microbiome [7].

In horses, the influence of diet and environmental factors on intestinal microbial composition is well documented [8,9]. Other factors such as geographic area, age, stress, and season also affect the gut microbiome in healthy horses [10,11]. Although there are differences in the relative abundance of different phyla, *Firmicuteota* (formerly *Firmicutes*), and *Bacteroidetes* represent up to 90% of the gut microbiome in horses [12,13,14,15,16]. These phyla, together with *Spirochaetes*, have been functionally linked to carbohydrate metabolism, energy metabolic processes, SCFA production, and lactic acid production [17]. Within *Bacillota*, *Lachnospiraceae* and *Ruminococcus* groups are abundant in racehorses and, along with *Oscillospiraceae*, appear to be associated with high sports performance [18,19].

As in humans, certain alterations in horse gut microbiome composition, or dysbiosis, have been linked to different diseases [2,20,21,22]. Some examples in horses are colitis, metabolic syndrome, and colic [15,23,24]. Colic in horses is a serious condition, and it is unclear whether alterations in the gut microbiome are a cause or a consequence in this pathology [25]. Colic in horses refers to abdominal pain and discomfort that can have various underlying causes, affecting the gastrointestinal tract [26]. It is a common and potentially serious condition, often requiring prompt veterinary attention. In horses with colic, a loss of bacterial richness and diversity has been reported [22,27,28]. In addition, a decrease in the relative abundance of *Bacillota* and *Bacteroidetes* and an increase in opportunistic pathogens such as *Bacillus* sp., *Streptococcus* sp., and members of the Enterobacteriaceae family have been reported [29]. It has been hypothesized that some types of colic may be related to the lack of adaptation of microbial communities to environmental factors such as highly concentrated diets, poor quality forage, stress, fasting, and antibiotics, among others [15,16,28,30,31,32,33], which could explain the variations observed between different groups [20,31].

Massive sequencing technologies can provide useful information regarding the microbial communities in the horse intestinal microbiome [23,25]. However, 16S rRNA profiling studies do not offer mechanistic insights because they do not consider the functional characteristics of the microbial communities involved. Understanding colic from a microbial functional perspective may serve as a basis for the development of preventive, diagnostic, and therapeutic interventions. This study aimed to compare the intestinal microbiome between healthy and colic horses using 16S rRNA sequencing and predict enriched metabolic functions in their microbiomes.

## 2. Materials and Methods

### 2.1. Ethics Statement

The research protocol was reviewed and approved by the University Andrés Bello Institutional Bioethics Committee (protocol number 008/2020). Horse owners voluntarily agreed to participate in this study and signed informed consent before sampling. The methods were performed according to approved guidelines.

### 2.2. Sampling Location

The Equine Veterinary Clinical Hospital (HCVE), belonging to University Andrés Bello, is in the Metropolitan Region, Chile (33°27′25″ S 70°38.896′ W). This hospital treats an average of 600 patients a year and historically receives horses of different breeds.

### 2.3. Criteria Inclusion

Twenty-eight adult horses of different breeds and sex admitted to the HCVE of Universidad Andrés Bello between August 2022 and January 2023 were included. All participants were correctly dewormed and vaccinated. Animals were included without antibiotic and corticosteroid treatment, at least 30 days before sampling [34,35]. The sample size was calculated using the formula for the difference between means, considering a level of error of 0.05 and a standard deviation of 2. The sampled group was divided into two groups. Group 1: Healthy animals (n = 14), composed of horses without underlying pathologies, who attended the hospital for reproductive or dental check-ups. Blood parameters were within normal ranges. Group 2: Animals with colic (n = 14) consisting of horses with colic due to large intestine involvement, diagnosis made by anamnesis, observation, physical examination, and transrectal palpation. In addition, transcutaneous abdominal ultrasound, percutaneous abdominocentesis, or exploratory laparotomy was performed, as appropriate for each patient. In this group of patients, the heart rate, respiratory rate, and lactate values were observed at the upper limit or outside the normal range, with averages of 61 (44–96) beats/min, 23 breaths/min, and 3.3 (2.5–4.7) mmol/L, respectively. In both groups, stool samples were collected using rectal swabs at the time the patient entered the hospital. The swabs were transported in Cary Blair media (Linsan, Santiago, Chile) and immediately processed in the clinical microbiology and microbiome laboratory (UNAB).

### 2.4. Microbiome Study

Fecal DNA was extracted using the Quick-DNA Fecal/Soil Microbe kit (Zymo Research, Irvine, CA, USA) according to the manufacturer’s instructions; a negative control was included. Samples were prepared using Zymo Quick-16S kit (Zymo Research, Irvine, CA, USA) with phased primers targeting the V3/V4 regions of the 16S gene. The specific primer sequences were (5′-CCTACGGGDGGCWGCAG-3′), (3′-GACTANVGGGTMTCTAATCC-5′) and (5′-CCTAYGGGGYGCWGCAG-3′), (3′-GACTACNVGGGTMTCTAATCC-5′), respectively. Following clean up and normalization, the samples were sequenced on a P1 600cyc NextSeq2000 Flow cell (Illumina, San Diego, CA, USA) to generate 2 × 301 bp paired end (PE) reads. Quality control and adapter trimming were performed with bcl-convert. The DNA sequences provided by the external service (Seq Center (Pittsburgh, PA, USA)) were analyzed using a bioinformatics pipeline that included the following steps: (1) sample demultiplexing (QIIME version 1.8.0, [36]), (2) denoising of sequences and taxonomic assignment (DADA2 version 1.2.2 and R version 4.1.2, [37]), (3) prediction of metabolic functions (PICRUSt2, [38]), and (4) statistical analyses (scipy v1.10.0, [39]; LEfSe version 1.1.2 [40]). First, the individual samples were extracted from the provided fastq file using the QIIME demultiplex_fasta.py script. Then, all samples were processed using the DADA2 version 1.22 software to remove low-quality reads, remove reads with indeterminate base calls, and trim them down to 220 nucleotides. Next, the reads were used to infer a sequencing error model, and the model was used to denoise the reads and obtain amplicon sequence variants (ASVs). Each ASV was assigned to a bacterial taxonomy employing a naïve Bayesian classifier [41] and the SILVA database version 138 [42,43], using the assign Taxonomy and add Species functions from DADA2. Any ASV with a low abundance (<0.1%) was removed from subsequent analyses. Finally, the abundance table of each ASV was used to infer the abundance of metabolic functions and pathways using the PICRUSt2 version 2.5.0 software [38] with the included MetaCyc database. Rarefaction curves were determined using the vegan software version 2.6-4 (https://cran.r-project.org/web/packages/vegan/index.html, accessed on 2 March 23).

### 2.5. Statistical Analyses

Statistical analyses were performed using the Mann–Whitney U test from the Python scipy package v1.10.0 [39] for the relative abundance of ASVs. Similarly, the LEfSe version 1.1.2 software [40] was used to determine significant differences in the relative abundance of metabolic functions and pathways. LEfSe version 1.1.2 was executed by normalizing the abundance of metabolic functions and pathways to one million per sample. A statistically significant difference for the Mann–Whitney U test was established at *p*-value < 0.05, and for the linear discriminant analysis (LDA) at log10 (LDA) greater or equal to 2.0. In addition, the weighted UniFrac method from the Python scikit-bio package version 0.5.8 (http://scikit-bio.org, accessed on 28 September 2024) was applied to the relative abundance at the phylum level, and a principal component analysis using the Python scikit-learn package version 1.1.2 [44] was performed for the abundance of functions and pathways.

### 2.6. Data Availability

The datasets generated during the current study are available at the European Nucleotide Archive repository, under the code PRJEB47719 (https://www.ebi.ac.uk/ena, accessed on 2 March 2023).

## 3. Results

Fecal samples were obtained from 14 healthy horses (control) and 14 horses with colic (Table 1). The mean age was 8.4 and 11.1 years for the control and colic groups, respectively. Most of the horses were of the Chilean breed, 64.3% in the control group and 57.1% in the colic group. Sex was uniformly distributed between groups (Table 1). Table 2 shows the characteristics of the group with colic, where we can see that 100% are associated with large intestine conditions and all of them were resolved surgically.

**Figure 1:**
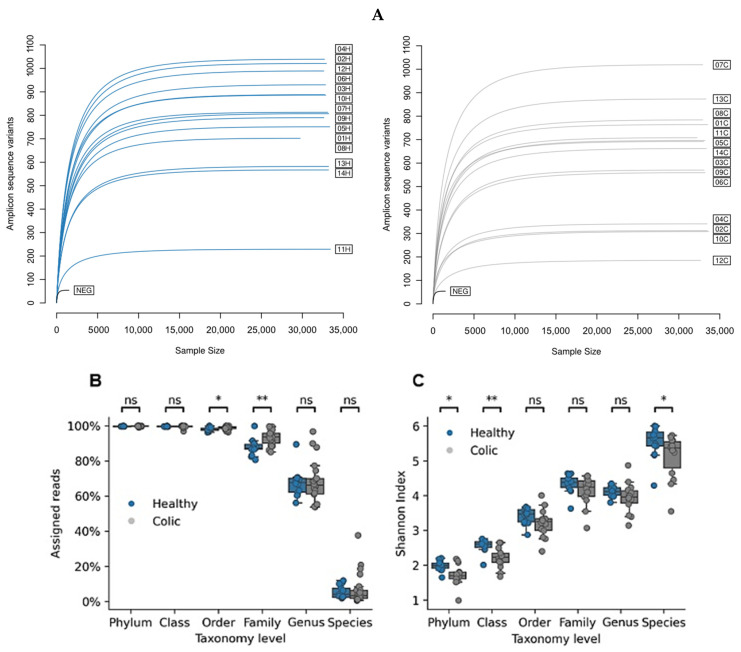
(**A**) Rarefaction curve and taxonomy assignment coverage. The abundance of each unique amplicon sequence variant (ASV) was determined at random sampling depths using the vegan software version 2.6-4. Dark blue lines, samples from healthy individuals. Light gray lines, samples from individuals diagnosed with colic. (**B**) The percentage of reads at different taxonomic levels that were classified using DADA2 and the SILVA database version 138. Dark blue dots, ASVs from healthy individuals; light gray, ASVs from individuals with a colic diagnosis. (**C**) Shannon index (alpha diversity) of total reads at different taxonomic levels. Unclassified reads replicate the last known taxonomic classification (e.g., “Unclassified species of genus *Alloprevotella*”). Dark blue dots, ASVs from healthy individuals; light gray, ASVs from individuals with a colic diagnosis. (*) = *p* value < 0.002; (**) = *p* value < 0.001 (ns) = not significant.

The fecal microbiome of healthy and colic horses was characterized using 16S rRNA sequencing. Rarefaction curves showed saturation in all samples, with a substantial variation in amplicon sequence variants (ASVs), ranging from nearly 200 to 1000 (Figure 1A). Read classification at each taxonomic level (Figure 1B) showed significant differences in the number of reads between healthy and colic groups at the species level, with the latter presenting fewer ASVs. In addition, alpha diversity measured by the Shannon index showed significant differences between groups at the phylum and class levels (Figure 1C), with reduced diversity in the colic group. As taxonomic assignment rates drop at the species level (Figure 1B), the diversity measured by the Shannon index lacks support.

**Figure 2:**
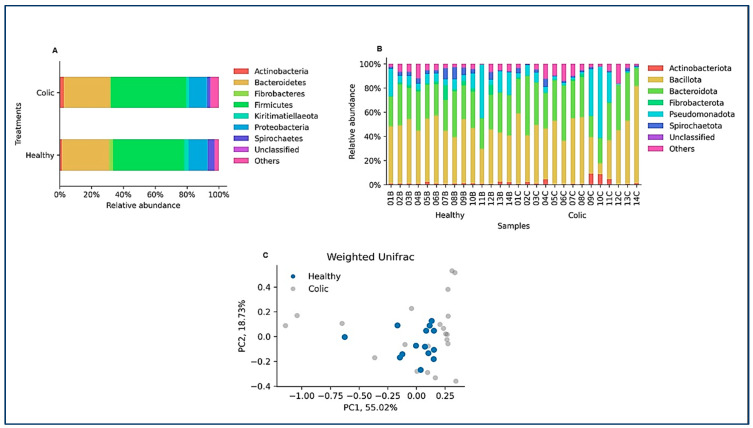
(**A**,**B**) The relative abundance of each phylum is averaged over all the samples per group and per sample. The “others” category groups the relative abundance of amplicon sequence variants that are represented at an average frequency of less than 1% per group. Samples from healthy animals are denoted as (**B**), while samples from colicky horses as (**C**). (**C**) A weighted UniFrac decomposition of the data shows extensive dispersion in the group of individuals with a colic diagnosis, overlapping with the healthy group. Dark blue dots, samples from healthy individuals; light gray, samples from individuals with a colic diagnosis.

**Figure 3:**
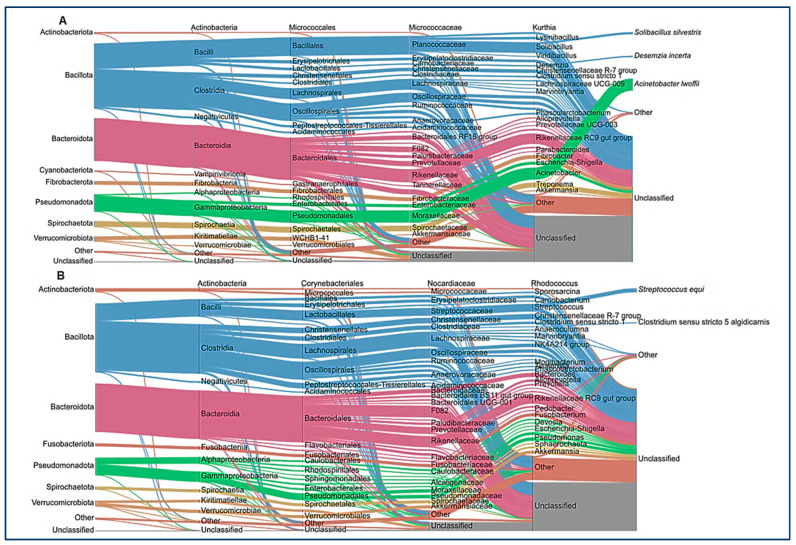
Average of the relative abundance at each taxonomic level. Sankey plots for each group ((**A**): healthy, (**B**): colic) show *Bacillota*, *Bacteroidota*, and *Pseudomonadota* as dominant phyla in both groups.

After ASV assignment, we analyzed the relative abundance at the phylum level in the animals of the study (Figure 2A). No significant differences between the healthy and colic groups were observed at the taxonomic level. Individual horse microbiome profiles were dominated by *Bacillota*, *Bacteroidota*, and *Pseudomonadota* (Figure 2B). Weighted UniFrac showed extensive dispersion of the microbiomes of colic horses, compared to healthy animals, which showed more similar microbiome compositions (Figure 2C). Finally, microbiome comparisons at every taxonomic level are shown in Figure 3. Both groups were characterized by similar abundances of *Rikenellaceae*, Lachnospiraceae, and *Oscillospiraceae*. Healthy horses showed higher abundances of *Solibacillus (Bacillales*), Fibrobacter (Fibrobacteriaceae), and *Acinetobacter (Moraxellaceae*), and the colic group showed an increase in *Streptococcus* (*Lactobacillaceae*; Figure 3).

**Figure 4:**
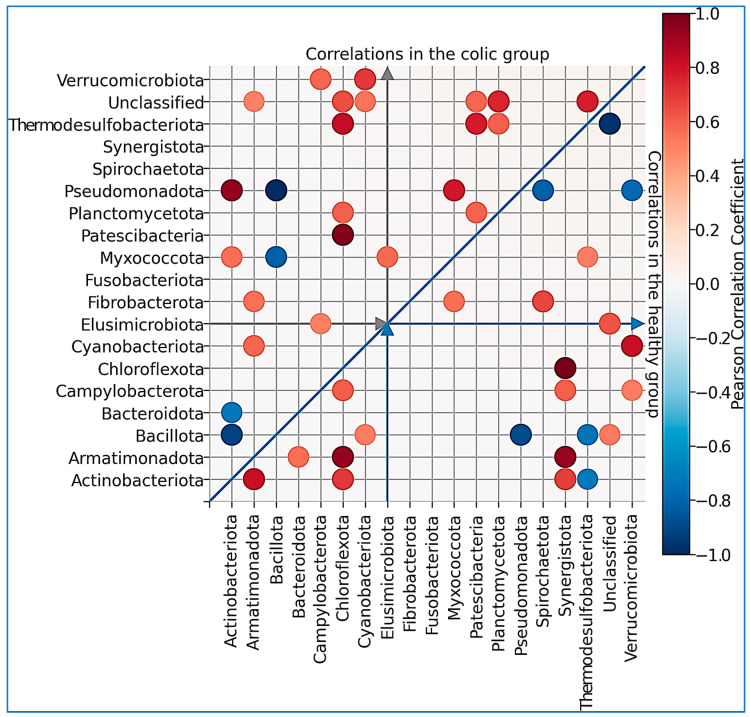
Pearson’s correlation coefficients within the group of healthy individuals (lower triangle) and within the group of individuals with a colic diagnosis (upper triangle) for significant values (*p*-value < 0.05).

We later searched for significant correlations between phyla among animals (Figure 4). The *Bacillota* phylum showed a negative correlation with *Pseudomonadota* in the healthy group. Similarly, in colic horses, *Bacillota* correlated negatively with *Actinobacteriota* and *Pseudomonadota*, and these two phyla correlated positively in this group (Figure 4).

**Figure 5:**
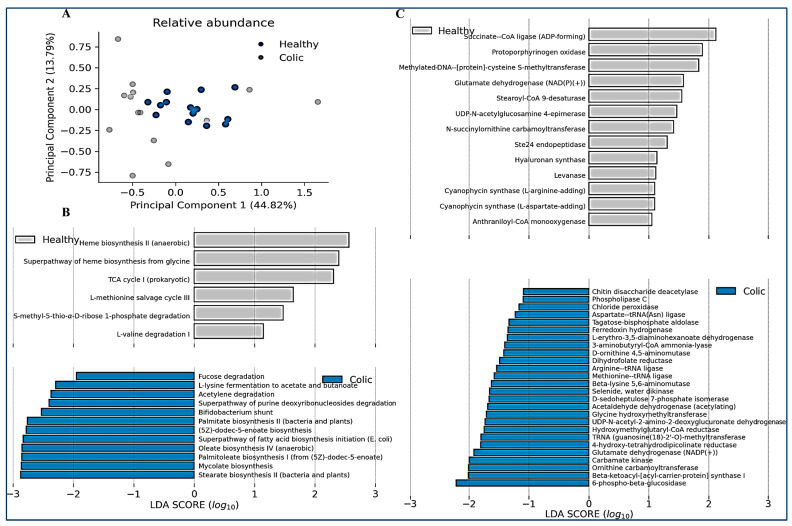
(**A**) Relative abundance of metabolic functions and linear discriminant analysis. A principal component analysis is shown considering all the relative abundances of metabolic functions. (**B**) The relative abundance of pathways for each sampled individual was determined using the PICRUSt2 version 2.5.0 software and is shown for every pathway with a significant LDA score greater than 1.0 (right, scatter plot). (**C**) The relative abundance of functions for every sample was determined using the PICRUSt2 version 2.5.0 software and is shown for every function with a significant LDA score over 1.0 (right, scatter plot).

We finally compared the relative abundance of the predicted metabolic functions and metabolic pathways in both groups of animals (Figure 5). Like microbiome composition PCA analysis, an important variation in the predicted functions in the colic group was observed, compared with control animals, indicating they have different functionalities (Figure 5A). Metabolic pathways enriched in the control group, but reduced in colic horses, were associated with heme biosynthesis and the TCA (tricarboxylic acid) cycle (Figure 5B). This correlated with enrichment in metabolic functions such as succinate–CoA ligase and protoporphynogen oxidase (Figure 5C). In contrast, colic horses were predicted to be enriched in pathways such as fatty acid biosynthesis (palmitate, stearate, oleate, and palmitoleate), the bifid shunt (characteristic of Actinobacteria and Bifidobacterium), and degradation of metabolites such as lysine, purines, fucose, and acetylene (Figure 5B). Other functions increased in colic horses were related to arginine degradation and 6-phospho-beta-glucosidase (Figure 5C).

## 4. Discussion

Colic in horses is a potentially fatal condition that has been associated with changes in the gut microbiome [29]. The equine gut microbiome is an understudied microbial community that might impact horse health [45]. Here, we found discrete microbiological changes in the gut microbiome of horses with colic, which were associated with a more aerobic metabolism. Colic in horses could be associated with multiple underlying causes [46]. Therefore, changes in gut bacteria associated with colic might be influenced by different factors beyond colic, posing a limitation to our interpretations.

In addition, although the selected cases of the group of patients with colic were associated with a condition of the large intestine, we do not have clear information on the management that could have been carried out before they entered the hospital, since many of them are treated by their own owners or caregivers before admission. A total of 50% of the cases in this group were admitted due to colon impaction, so it is likely that they were subjected to dietary restriction or fluid therapy, information that should be considered as a limitation.

Like other studies, Chilean horses were characterized by a dominance of *Bacillota*, *Bacteroidetes*, and *Pseudomonadota* [14,23]. Several studies have shown significant changes in the equine gut microbiome in colic or other intestinal diseases [47]. Studies have shown that horses with intestinal diseases exhibit reduced microbial diversity and species richness compared to healthy horses [29,34]. Colic horses show reduced diversity [29], consistent with the findings of this study. Microbial diversity is a critical marker of the gut microbiome and reduction in diversity is usually accompanied by dysbiosis and the onset of clinical conditions [47,48].

In addition, we observed reduced levels of *Fibrobacter*, *Solibacillus*, and *Acinetobacter* in colic. *Fibrobacter* spp. are important cellulolytic bacteria in the hindgut of herbivores, including horses [49,50]. A reduction in their abundance might correlate with less fiber degradation in the colic horse hindgut [9]. The genus *Solibacillus* includes Gram-positive aerobic spore-forming bacteria, which appears to be decreased in induced laminitis in horses [51]. However, very few studies have addressed their relevance in the horse gut microbiota. Finally, *Acinetobacter* spp. have been studied in the context of antimicrobial resistance (AMR), where *A. baumanii* is found in horses and could play a role in AMR transmission [52,53,54].

Lactic acid bacteria appear to be an important bacterial group in the equine microbiome [55]. Colic-affected horses deploy an overgrowth of lactic acid bacteria, including *Streptococcus* and *Lactobacillaceae*, which may contribute to hindgut pH changes and altered fermentation patterns [34]. The enrichment of *Streptococcus* in this group correlates with the literature.

The prediction of metabolic pathways and functions provided clues regarding changes in the gut microbiome of colic horses. The reduction in heme biosynthesis and TCA cycle in these animals suggest a switch from aerobic to anaerobic conditions, considering heme TCA enzymes are required for aerobic respiration. These conditions correlate with the increase in facultative anaerobes such as *Streptococcus* spp. The increase in fatty acid biosynthesis, and the degradation of certain metabolites such as purines, lysine, arginine, and fucose, are important predictions that differentiate colic from healthy horses. Some of these alterations have been shown to correlate with dysbiosis in different animals, and contribute to a pro-inflammatory gut environment, which usually result in harmful molecules such as ammonia, hydrogen sulfide, and p-cresol, among others [56,57,58]. While these predictions arise from reference genomes, functional metagenomic analyses are required to corroborate these findings and to reveal whether the predicted functional characteristics vary across different clinical presentations of colic in horses.

## 5. Conclusions

This prospective study highlights distinct changes in the fecal microbiome and predicted the metabolic functions of horses with colic. Colic horses showed reduced microbial diversity and decreases in key bacterial groups like *Fibrobacter* and *Solibacillus*, consistent with gut dysbiosis. The overgrowth of lactic acid bacteria, particularly *Streptococcus*, suggests shifts in fermentation that may contribute to altered gut conditions. Metabolic predictions indicated reduced aerobic functions, such as heme biosynthesis and the TCA cycle, along with an increase in fatty acid biosynthesis and metabolite degradation. Determining the impact of these findings on colic prevention and management demands further investigation, differentiating small versus large intestinal disease and acute versus recurrent colic.

## Figures and Tables

**Table 1:** Epidemiological characteristics of the participants.

Category	Variable	Control (N°/%)	Colic (N°/%)
Age	Young (3–4 years)	3/21%	1/7.1%
	Mature (5–15 years)	10/71%	12/90%
	Geriatric (>15 years)	1/7%	1/7.1%
Breed	Chilean	9/64.3%	8/57.2%
	Warmblood	1/7.1%	4/28.5%
	Thoroughbred	3/21%	1/7.1%
	Quarter horse	1/7.1%	1/7.1%
Sex	Gelding	7/50%	7/50%
	Female	6/43%	4/30%
	Stallion	1/7%	3/20%

**Table 2:** Clinical characteristics of the group of horses with colic.

Diagnosis	Treatment Category	Number
Large colon impaction	Surgical	7
Large colon displacement	Surgical	2
Large intestinal rupture	Surgical	1
Large colon torsion	Surgical	4

## Data Availability

Publicly available datasets were analyzed in this study. These data can be found here: https://www.ebi.ac.uk/ena/browser/view/PRJEB47719, accessed on 28 September 2024.
